# A novel generation 1928zT2 CAR T cells induce remission in extramedullary relapse of acute lymphoblastic leukemia

**DOI:** 10.1186/s13045-018-0572-x

**Published:** 2018-02-20

**Authors:** Jianyu Weng, Peilong Lai, Le Qin, Yunxin Lai, Zhiwu Jiang, Chenwei Luo, Xin Huang, Suijing Wu, Dan Shao, Chengxin Deng, Lisi Huang, Zesheng Lu, Maohua Zhou, Lingji Zeng, Dongmei Chen, Yulian Wang, Xiaomei Chen, Suxia Geng, Weinkove Robert, Zhaoyang Tang, Chang He, Peng Li, Xin Du

**Affiliations:** 1 Department of Hematology, Guangdong General Hospital, Guangdong Academy of Medical Sciences, Guangzhou, 510080 China; 2 Key Laboratory of Regenerative Biology, South China Institute for Stem Cell Biology and Regenerative Medicine, Guangzhou Institutes of Biomedicine and Health, Chinese Academy of Sciences, Guangzhou, 510530 China; 3 Guangdong Provincial Key Laboratory of Stem Cell and Regenerative Medicine, South China Institute for Stem Cell Biology and Regenerative Medicine, Guangzhou Institutes of Biomedicine and Health, Chinese Academy of Sciences, Guangzhou, 510530 China; 4 Department of PET Center, Guangdong General Hospital and Guangdong Academy of Medical Sciences, Guangzhou, 510080 China; 5 Wellington Blood and Cancer Centre, Capital and Coast District Health Board, Wellington, New Zealand; 6 Guangdong Zhaotai InVivo Biomedicine Co. Ltd., Guangzhou, 510000 China; 7 Hunan Zhaotai Yongren Medical Innovation Co. Ltd., Changsha, 410000 China; 8 State Key Laboratory of Ophthalmology, Zhongshan Ophthalmic Center, Sun Yat-Sen University, Guangzhou, 510060 People’s Republic of China

**Keywords:** Chimeric antigen receptor (CAR) T cells, Acute lymphocytic leukemia (ALL), Extramedullary ALL, Relapsed and refractory, TLR2

## Abstract

**Background:**

Anti-CD19 chimeric antigen receptor (CAR) T cells have shown promise in the treatment of B cell acute lymphocytic leukemia (B-ALL). However, its efficacy in B-ALL patients with extramedullary involvement is limited due to poor responses and neurotoxicity. Here, we utilized a third generation of CAR T cell vector, which contains the Toll/interleukin-1 receptor (ITR) domain of Toll-like receptor 2 (TLR2), to generate 1928zT2 T cells targeting CD19, and evaluated the efficacy of 1928zT2 T cells in relapse or refractory B-ALL patients with extramedullary involvement.

**Methods:**

1928zT2 T cells were generated by 19-28z-TLR2 lentiviral vector transfection into primary human T lymphocytes. The anti-leukemia effect of 1928zT2 T cells were determined by killing assays and in xenografts. Three patients diagnosed as relapse or refractory ALL with extramedullary involvement were infused with 1928zT2 T cells, and the clinical responses were evaluated by BM smear, B-ultrasonography, PET/CT, histology, flow cytometry, qPCR, ELISA, and luminex assay.

**Results:**

1928zT2 T cells exhibited enhanced effector function against CD19+ leukemic cells in vitro and in a xenograft model of human extramedullary leukemia. Notably, the 1928zT2 T cells eradicated extramedullary leukemia and induced complete remission in the three relapse and refractory ALL patients without serious adverse effects. 1928zT2 T cells expanded robustly in the circulation of these three patients and were detected in the cerebrospinal fluid of patient 3. These three patients experienced cytokine release syndrome (CRS) with grade 2 or 3, which remitted spontaneously or after tocilizumab treatment. None of the three patients suffered neurotoxicity or needed further intensive care.

**Conclusions:**

Our results demonstrate that 1928zT2 T cells with TLR2 incorporation augment anti-leukemic effects, particularly for eradicating extramedullary leukemia cells, and suggest that the infusion of 1928zT2 T cells is an encouraging treatment for relapsed/refractory ALL patients with extramedullary involvement.

**Trial registration:**

ClinicalTrials.gov identifier NCT02822326. Date of registration: July 4, 2016.

## Background

Despite great advances, relapsed and refractory B cell acute lymphoblastic leukemia (B-ALL) shows unfavorable prognosis, especially for adult patients [1, 2]. Although newly diagnosed ALL patients co-occurring with extramedullary involvement are rare, ALL with extramedullary tissues (EM-ALL) occurs in about 15 to 20% of all the relapse patients [3]. The central nervous system (CNS) is a favorable site for ALL relapse, accounting for two thirds of extramedullary relapse [4]. Besides, the extramedullary leukemia appears in the skin, breasts, bones, muscle, abdominal organs, and eyes. The presence of extramedullary leukemia is often associated with significantly shorter progression-free survival and overall survival [5]. Worse yet, there is no standard management for these patients and they have to confront dismal outcomes.

Chimeric antigen receptor (CAR) T cells are emerging as a powerful therapy for relapse or refractory ALL [6, 7]; however, the conventional CAR-T cells had shown little evidence of antitumor activity against extramedullary leukemia [8]. Moreover, the efficacy of CAR T cells against solid tumors is substantially poorer than that against leukemia [9, 10], impelling us to improve the design of CAR T vectors. The leukemia infiltrated into extramedullary tissues is often associated with poor response to therapy [8]. To our knowledge, most previous clinical trials of CAR T cells treatment did not enroll the patients with extramedullary leukemia [11].

Recently, we generated 1928zT2 T cells targeting CD19 by introducing the Toll/interleukin-1 receptor (TIR) domain of Toll-like receptor 2 (TLR2) to 1928z [12]. The activation of TLR2, a canonical costimulatory receptor, promotes the regression of established leukemia [13]. In addition, incorporation with TLR2 domain improves expansion of CAR-T cells and potentiates the killing capacity of CAR T cells against CD19^+^ leukemic cells [12]. Actually, the leukemia cells with potent abilities for cell migration, proliferation, adhesion, and protease activity facilitated themselves extramedullary infiltration [3, 4]. In this context, it is highly warranted to find tumor-specific T cells with enhanced activity against the leukemic cells of high affinity for the extramedullary relapse. The advent of novel 1928zT2 T cells with improved expansion and enhanced anti-tumor function causes it hopeful to treat such a complicated and aggressive disorder.

In this study, we report the remarkable ability of 1928zT2 T cells to induce complete remission (CR) in extramedullary tissues of three ALL patients, who had a relapse with resistant to available therapies. Our results provide evidences of extensive usage of CAR T cells in these adults, who have aggressive and often fatal relapsed ALL with extramedullary involvement.

## Methods

### Patient enrollment and characteristics

The trial (ClinicalTrials.gov number, NCT02822326) was designed to assess the safety and feasibility of infusing 1928zT2 T cells in patients with relapsed or refractory B-ALL and was approved by the Ethics Committee of Guangdong General Hospital. Here, we reported the first three enrolled ALL patients with extramedullary involvement (Table 1).

**Table 1:** The characteristics of enrolled ALL patients with extramedullary involvement

Patient no.	Age (years)	Sex	Recurrent site	Dose*	Response	CRS	allo-HSCT	Outcome^#^
No.1	34	F	Breast	0.5	CR on day 46	Grade 2	No	CR
No.2	16	M	BM, kidney	5.0	CR on day 10	Grade 2	Yes	CR
No.3	20	M	CNS, pancreas, pleuroperitoneum, pelvic fascia, and LNs	10	CR on day 18	Grade 3	No	CR

*M* male, *F* female, *CR* complete remission, *allo-HSCT* allogeneic hematopoietic stem cell transplantation, *CRS* severe cytokine release syndrome, *BM* bone marrow, *CNS* central nervous system; LNs, lymph nodes

*Dose at × 10^5^cells/kg

^#^Outcome in October 2017

Patient 1 was a 34-year-old female diagnosed as B-ALL (CD19+, BCR/ABL-) in April, 2015 (Fig. 3a). Although she had no response to chemotherapy regimen of VDLCP at first, the patient achieved CR after Hyper CVAD A therapy. She received four cycles of chemotherapy and underwent allogeneic hematopoietic stem cell transplantation (allo-HSCT) from her 10/10 HLA allele-matched sister in November, 2015. However, 9 months later, she had a relapse in extramedullary tissues including her left breast and multiple lymph nodes identified by Positron emission tomography-computed tomography (PET/CT) (Fig. 3b). The extramedullary leukemia in breast was confirmed histologically (Fig. 3c), and leukemia blast cells were detected as positive for TdT, CD19, CD20, CD79a, CD34, CD99, CD10, PAX5, and Ki67 (15%), and negative for CD3 and Cyclin D1. B-mode ultrasound was used to monitor the tumor mass in the left breast, and about 2.8 × 1.6 cm size of an inhomogeneous hypo-echoic mass was identified (Fig. 3d). No evidence of relapse in BM and CNS was observed with persisted complete donor chimerism or negative minimal residual disease.

Patient 2 was a 15-year-old male also diagnosed as B-ALL (CD19+, BCR/ABL-) in October, 2014 (Fig. 4a). He underwent allo-HSCT from his 10/10 HLA allele-matched sibling in June, 2015, and unfortunately had a relapse in CNS 6 months later. Then, he achieved a second CR after intrathecal chemotherapy (IT), irradiation, and donor lymphocyte infusion (DLI). However, the leukemia recurred again 10 months later with BM and extramedullary involvement. The BM smear showed typical leukemic blasts account for 15% (Fig. 4b). The PET/CT revealed that an abnormal intense high metabolic region beneath the kidney capsule, indicating the extramedullary relapse in kidney (Fig. 4c). Although he received chemotherapy regimen of VDLCP, he had no response at all.

Patient 3 was a 20-year-old male with a third recurrence of ALL with CNS-3 status [14] and extramedullary involvement. He received a diagnosis of ALL (CD19^+^, BCR/ABL-) in 2000 when he was 3 years old (Fig. 5a). During the following 13 years, he had received more than 20 courses of intensive systemic chemotherapy (SC) and IT and remained being in CR. However, he had a relapse in BM and CNS in 2013. Then, he underwent allo-HSCT from a 10/10 HLA allele-matched unrelated female donor in 2014 and the second CR was achieved. Despite he received IT for more than six courses, the patient suffered from second recurrence with CNS-ALL. He had a good response to IT and irradiation and achieved CR for a third time. However, the cancer recurred again 10 months later with CNS and extramedullary involvement in June, 2017. The bone marrow examination still showed signs of complete remission. The whole-body PET/CT showed that extensive extramedullary relapse involving almost the entire pleura, peritoneum, pelvic fascia, pancreas, and multiple lymph nodes (Fig. 5b). A biopsy was performed on right-sided supraclavicular node mass, and characteristic megakaryocytes, erythroblasts and myeloid cells were observed microscopically. The markers were identified as CD3−, CD20−, CD19+, TdT+++, CD10+++, CD34−, and Ki67+ (90%), revealing the leukemic involvement (Fig. 5c). A palpable mass below xiphoid was identified by B-mode ultrasound, and inhomogeneous hypo-echoic mass about 3.0 × 1.9 cm in diameter was seen (Fig. 5d). In addition, aberrant blast cells were detected histologically (Fig. 5e) and examined CD19^+^CD10^+^ phenotype (Fig. 5f) in the CSF obtained by lumbar puncture. X/Y fluorescent in situ hybridization (FISH) showed that XY-type recipient cells accounted for 50% of the cerebrospinal cells, further indicating a relapse in the CSF (Fig. 5g).

### Clinical protocols

Patients underwent an apheresis to obtain sufficient PBMCs for producing 1928zT2 T cells. The manufacture of 1928zT2 was completed in about 14 days. The cells were released for infusion after safety check. All patients received a lymphodepletion conditioning regimen which contained with fludarabine 25 mg/m^2^, cyclophosphamide 300 mg/m^2^, and/or plus other drugs. After 1-day rest, they received a split-dose infusion of 1928zT2 T cells (a total of 5 × 10^4^–1 × 10^6^ cells/kg and 10% on day 1, 30% on day 2, and 60% on day 3). No post-infusion cytokines such as IL-2 were administered. These patients were evaluated for responses and toxic effects. The expansion and persistence of circulating 1928zT2 T cells were also monitored. Adverse events such as CRS after 1928zT2 T cell infusion were graded according to National Institutes of Health criteria (Common terminology Criteria for Adverse Events, version 4) [8], and clinical responses were evaluated and defined as complete remission (CR), CR with incomplete count recovery (CRi), partial response (PR), stable disease (SD), or progressive disease (PD) according with the National Comprehensive Cancer Network (NCCN) guidelines. MRD negative was defined as the absence of leukemia cells in BM determined by flow cytometry, and the absence of fusion gene in BM determined by qPCR.

### Generation and expansion of 1928zT2 T cells

The 1928zT2 T cells were generated as previously described [14]. Briefly, the 19-28z-TLR2 lentiviral vector was established with 19-28z-TLR2, a CAR-linking FMC63-scFv, CD28 transmembrane and endodomain, the CD3ζ signaling domain, and the TIR domain (amino acid 639-784) of TLR2. Primary human T lymphocytes were isolated and collected, then stimulated with magnetic beads coated with anti-CD3/anti-CD28 antibodies (Miltenyi Biotec) at cell to bead ratio of 1 to 2. Approximately 72 h after activation, T cells were transfected with 19-28z-TLR2 lentiviral vector. These T cells were fed every 2 days with fresh media and used within 14 days of expansion.

### Flow cytometry of CD19-CAR T cells detection

The percentages of transduced GFP-positive T cells, CD19 on leukemia cells, T cell phenotypes of the patient were determined by flow cytometry. The antibodies for CD3 (APC), CD4 (Percp-cy5.5), CD8 (PE), CD19 (APC), CD20 (PE), and CD22 (Percp-cy5.5) were used. The flow cytometry was performed with a FACSCalibur flow cytometer (BD Biosciences) and analyzed using FlowJo software.

### Killing assays

The target cells K562, K562-CD19, and NALM6 were tagged both GFP and luciferase (GL), specifically incubated with GFP T, 1928z T or 1928zT2 T cells at the indicated ratios in triplicate wells in U-bottomed 96-well plates. Viability of target cells was monitored 18 h later by adding 100 dl/well substrate D-luciferin firefly (Life Sciences) resolved at 150 μg/ml. Background luminescence was negligible (< 1% than the signal from the wells with only target cells). The viability percentage was calculated as experimental signal/maximal signal × 100%, and killing percentage was equal to 100 − viability percentage.

### Xenograft models and in vivo assessment

To develop the mouse subcutaneous tumor xenograft model of human extramedullary ALL leukemia [15], NSI mice of ages 6 to 10 weeks were used. All procedures were performed under approval of the Institutional Animal Care and Use Ethics Committee. NALM6-GL (2 × 10^5^ cells in 200 μl PBS) was injected subcutaneously, and 2 × 10^6^ lentiviral transduced human T cells in 200 μl PBS were adoptively transferred to these mice systemically by tail vein injection 2 days later. In vivo whole body imaging of luciferase-labeled cells was performed by cooled CCD camera system (IVIS 100 Series Imaging System, Xenogen, Alameda, CA). D-Luciferin firefly, potassium salt (75 mg/kg) was injected, and the mice were imaged. Quantification of total and average emissions was performed using the Living Image software (Xenogen). The mice were killed at day 34, and their subcutaneous tumor masses were extracted and weighted.

### Cytokine measurements and luminex technology

IL-6 levels in plasma were assessed using enzyme-linked immunosorbent assay (ELISA kit, eBioscience, USA) according to the manufacturer’s instructions. The immune-related cytokines were analyzed using Luminex MAGPIX system (Luminex Corp., Austin, TX) according to the manufacturers’ specifications. Samples were detected in triplicate relative to standards supplied by the manufacturer and analyzed for significant differences between different groups.

### Quantitative real-time PCR

mRNA was extracted from cells with Trizol (Qiagen) and reverse transcribed into cDNA using the PrimeScript™ RT reagent Kit (Takara). All reactions were performed with TransStart Tip Green qPCR SuperMix (Transgene) on a Bio-Rad CFX96 real-time PCR machine. Delta CT calculations were relative to β-actin and were corrected for PCR efficiencies.

### Statistics

Statistical analysis was performed with SPSS software version 19.0 (Inc., Chicago, IL, USA). Student’s *t* test (two-tailed) or one-way analysis of variance was used, and data were presented as means ± SEM. A *P* value < 0.05 was considered significant.

## Results

### 1928zT2 T cells exhibited potent anti-leukemia effect in vitro

**Fig. 1:**
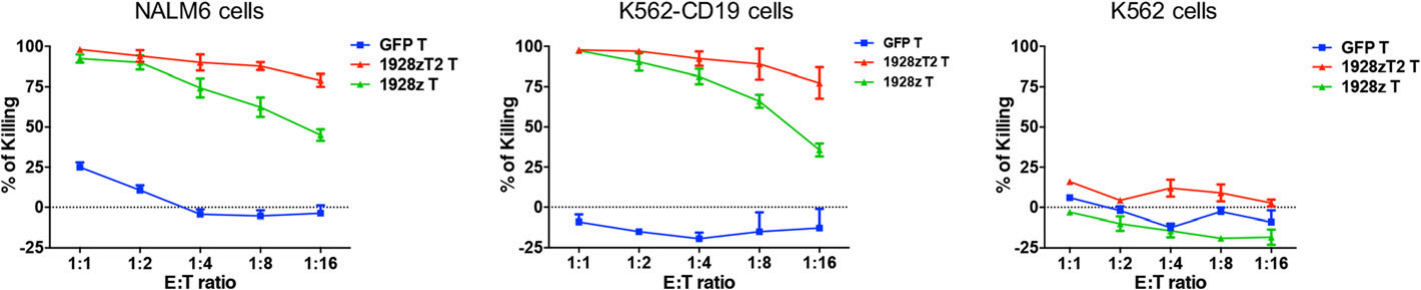
The 1928zT2 T cells presented elevated killing percentage of CD19+ cells compared with 1928zT cells in vitro. The killing assay with co-cultured 1928zT2 T cells and CD19-expressing NALM6 cells revealed that the 1928zT2 T cells lysed robustly the NALM6 cells at varied indicated ratios of E:T, in particular still eradicated more than 75% NALM6 cells at low ratios, whereas the 1928zT cells presented less than 50%. Similar data were observed in the K562 cells with CD19 expression, which were also utilized to evaluate the anti-CD19+ leukemia effect of 1928zT2 T cells. The K562 cells without CD19 expression were used as negative controls and virtually no killing effect was observed in 1928zT cells and GFP-control T cells

We firstly evaluated the in vitro cytotoxic responses of 1928zT2 T cells exposed to NALM-6 cells (a CD19+ human B-ALL cell line), K562-GL cells with or without CD19 expression. As showed in the Fig. 1, the 1928zT2 T cells generated from PBMC of ALL patient lysed more than 75% CD19-expressing NALM-6 cells at all indicated E:T ratios from 1:1 to 1:16, whereas less than 50% NALM-6 target cells were killed by 1928z T cells at E:T ratio of 1:16, indicating that the cytotoxicity of 1928zT2 T cells against target cells is more potent than that of 1928z T cells. Consistently, the results of killing assay with K562 cells revealed that both 1928z T and 1928zT2 T cells specifically killed the K562-CD19 cells but not the K562 cells without CD19 expression. Notably, the killing percentages of 1928zT2 T cells were higher than those of 1928z T cells at low E:T ratios. Thus, the 1928zT2 T cells exhibited enhanced anti-leukemic effect in vitro.

### TLR2 incorporation promoted the anti-leukemia effect of 1928zT2 T cells ex vivo

**Fig. 2:**
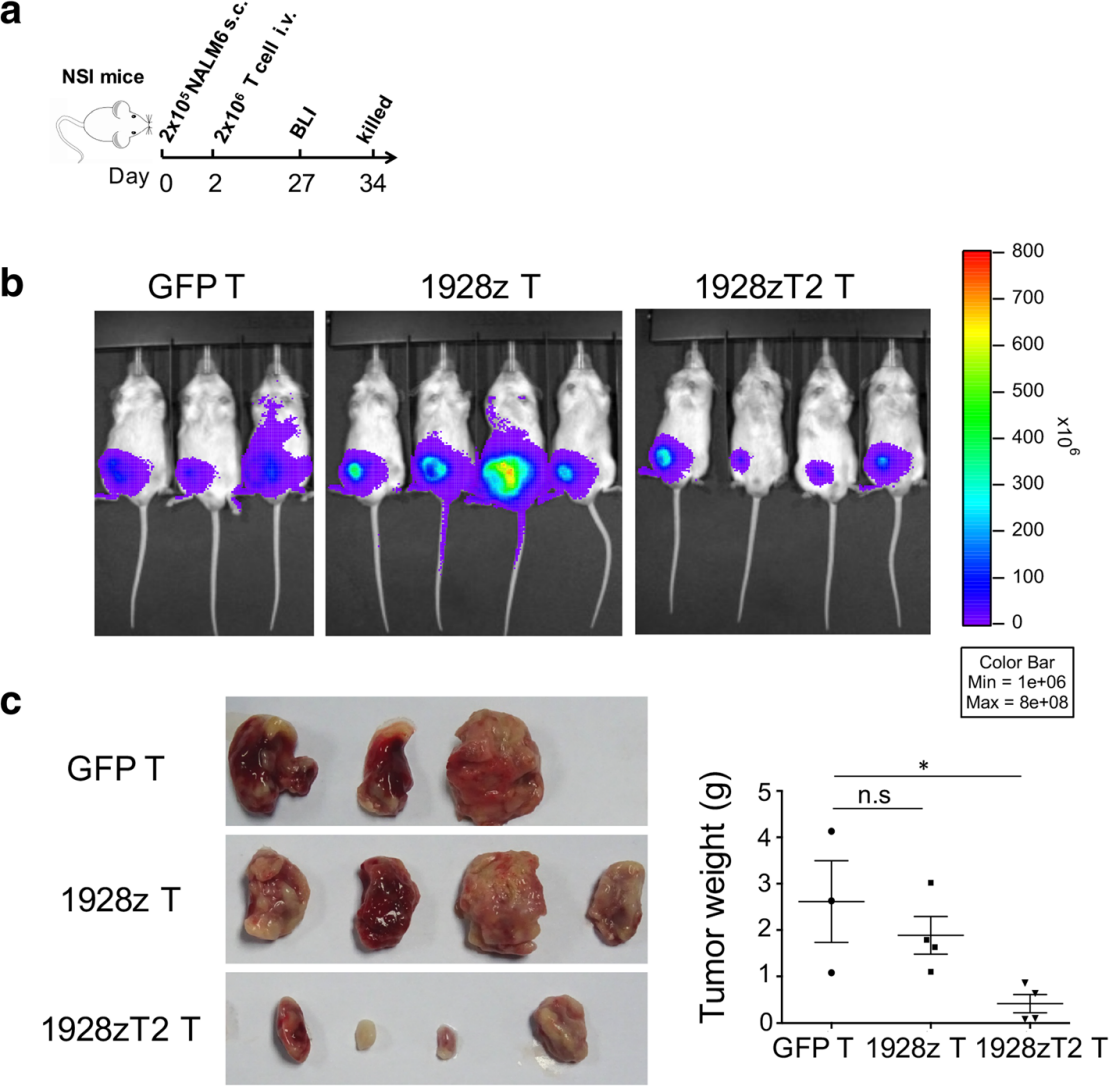
The 1928zT2 T cells promoted the regression of subcutaneous leukemia ex vivo. **a** The cell line-derived xenografts were established in the immunodeficient NSI mice by subcutaneous injection of 2 × 10^5^ NALM-6 cells. These mice received one infusion of 2 × 10^6^ GFP T, 1928z T cells, or 1928zT2 T cells, respectively. **b** BLI results revealed a marked tumor mass grew subcutaneously in the GFP-T group and similar signal was seen in the 1928z T cells group. However, the 1928zT2 T cells group exhibited significantly reduced signal compared to the other groups, indicating that tumors were significantly suppressed in these mice. **c** The tumor was extracted, and the specimen were showed. The tumor mass was smallest in the mice treated with 1928zT2 T cells, presenting as the lowest specific weight of tumor mass

To further address the potential therapeutic application of 1928zT2 T cells in extramedullary leukemia, we next examined whether these cells harbored cytolytic activity against subcutaneous leukemia ex vivo*.* The cell line-derived xenografts were established in the immunodeficient NSI mice by subcutaneous injection of 2 × 10^5^ NALM-6 cells that were tagged with GFP and luciferase. Two days later, these xenografts mice were administrated with one intravenous infusion of 2 × 10^6^ GFP T, 1928z T cells or 1928zT2 T cells (Fig. 2a). The BLI results revealed that tumors were significantly suppressed in mice treated with 1928zT2 T cells compared with those received GFP T cells (Fig. 2b), highlighting the potent efficacy of these novel 1928zT2 T cells against extramedullary leukemia. Conversely, there was no significant difference of tumor burden between 1928z T cells and GFP T cells. The tumors from mice that were treated with 1928zT2 T cells were smaller than that from control groups (Fig. 2c). Collectively, 1928zT2 T cells significantly reduced tumor burden in subcutaneous leukemia-bearing xenografts.

### Clinical responses to infusion of 1928zT2 T cells

**Fig. 3:**
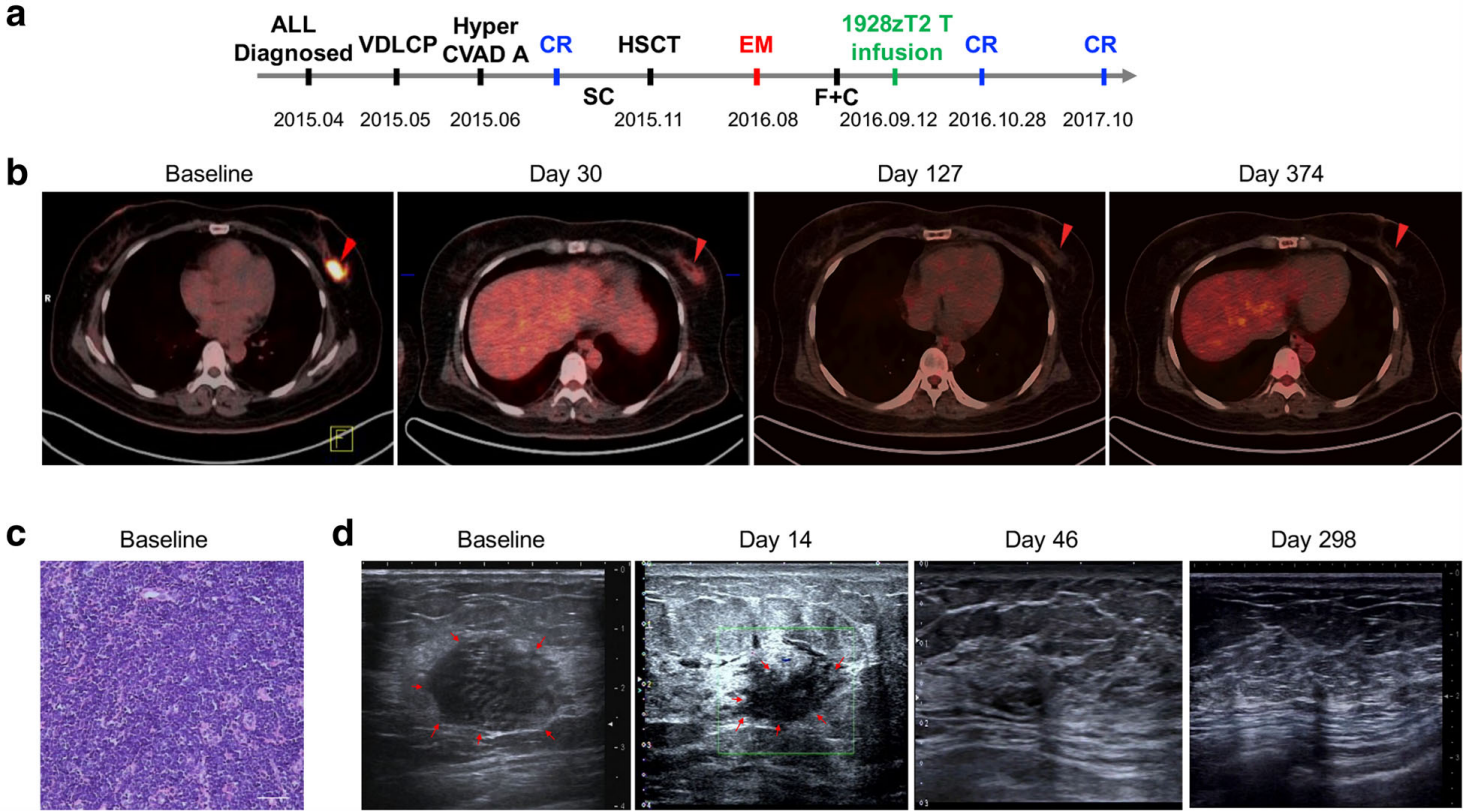
Small dose of 1928zT2 T cell infusion eradicated leukemia and induced CR in patient 1. **a** The diagram shows the development and therapeutic process of this ALL patient with extramedullary involvement. The 34-year-old female patient was diagnosed as B-ALL (CD19+, BCR/ABL-) in April, 2015, received allo-HSCT in November, 2015, and had a relapse in extramedullary (EM) tissues in August, 2016. She received fludarabine (F) and cytarabine (C) before cells infusion. Forty-six days after 1928zT2 T cells infusion (as low as 5 × 10^4^ cells/kg), the patient achieved CR and maintained remission in the follow-up. VDLCP, vincristine, daunomycin, cyclophosphamide, asparaginase, and dexamethasone; Hyper-CVAD A, cyclophosphamide, vincristine, doxorubicin, and dexamethasone; SC, systemic chemotherapy; **b** PET/CT data showed obviously an abnormal intense high metabolic mass in the left breast. Restage of PET/CT on day 30 after cells infusion presented that the lesion became hypometabolic state and no abnormal signal was observed thereafter. **c** The histological results showed the infiltration of megakaryocytes, erythroblasts, and myeloid cells in the tumor section, proven to be extramedullary relapse. **d** B-mode ultrasound showed an inhomogeneous hypo-echoic mass about 2.8 × 1.6 cm in diameter before cells infusion and reduction of mass size with 2.3 × 1.1 cm on day 14. The abnormal hypo-echoic mass was disappeared on day 46 and thereafter

The infusion of 1928zT2 T cells was well tolerated without any immediate adverse effects in these three patients. Strikingly, the size and hypometabolism of the first patient’s breast lump was reduced obviously, detected by PET/CT scanning on day 30 after 1928zT2 T cells infusion (Fig. 3b). The patient achieved complete remission on day 46 post infusion. Strikingly, the B ultrasound results showed that her breast lump disappeared compared to the baseline (Fig. 3d). PET/CT and B ultrasound restaging exhibited no evidence of relapse at all during the 12-month follow-up (Fig. 3b, d).

**Fig. 4:**
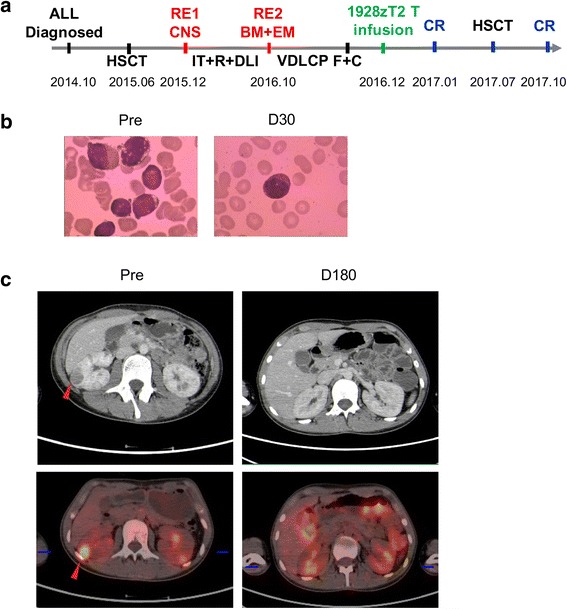
Infusion of 1928zT2 T cells induced complete remission in BM and extramedullary relapse in patient 2. **a** The 15-year-old male was diagnosed as B-ALL in October, 2014. He had the first recurrence (RE1) in CNS, and the leukemia recurred again with BM and extramedullary involvement (RE2). After 3-day usage of F and C, he received 1928zT2 T cells infusion (5 × 10^5^ cells/kg) and got CR 1 month later. Particularly, he underwent allo-HSCT again in July, 2017 and maintained remission during the follow-up. **b** The BM smear image revealed typical leukemia blasts at baseline disappeared 1 month after 1928zT2 T cells infusion. **c** The PET/CT revealed that an abnormal low density area and corresponding intense high metabolic region beneath the kidney capsule (red arrow). Restage of PET/CT after cells infusion presented that no low density area in the kidney, whereas there were diffuse high metabolic distribution with increased SUV due to the metabolism of 18F-FDG in the kidney

Patient 2 also had a complete remission in BM with no blast cells were observed in the BM smear 10 days after 1928zT2 T cells infusion (Fig. 4b). Six months later, restaging PET/CT revealed the absence of the abnormal intense high metabolic region, suggesting a remission in the kidney (Fig. 4c). This patient remained being in CR and received the second allo-HSCT from an unrelated donor in April 2017.

**Fig. 5:**
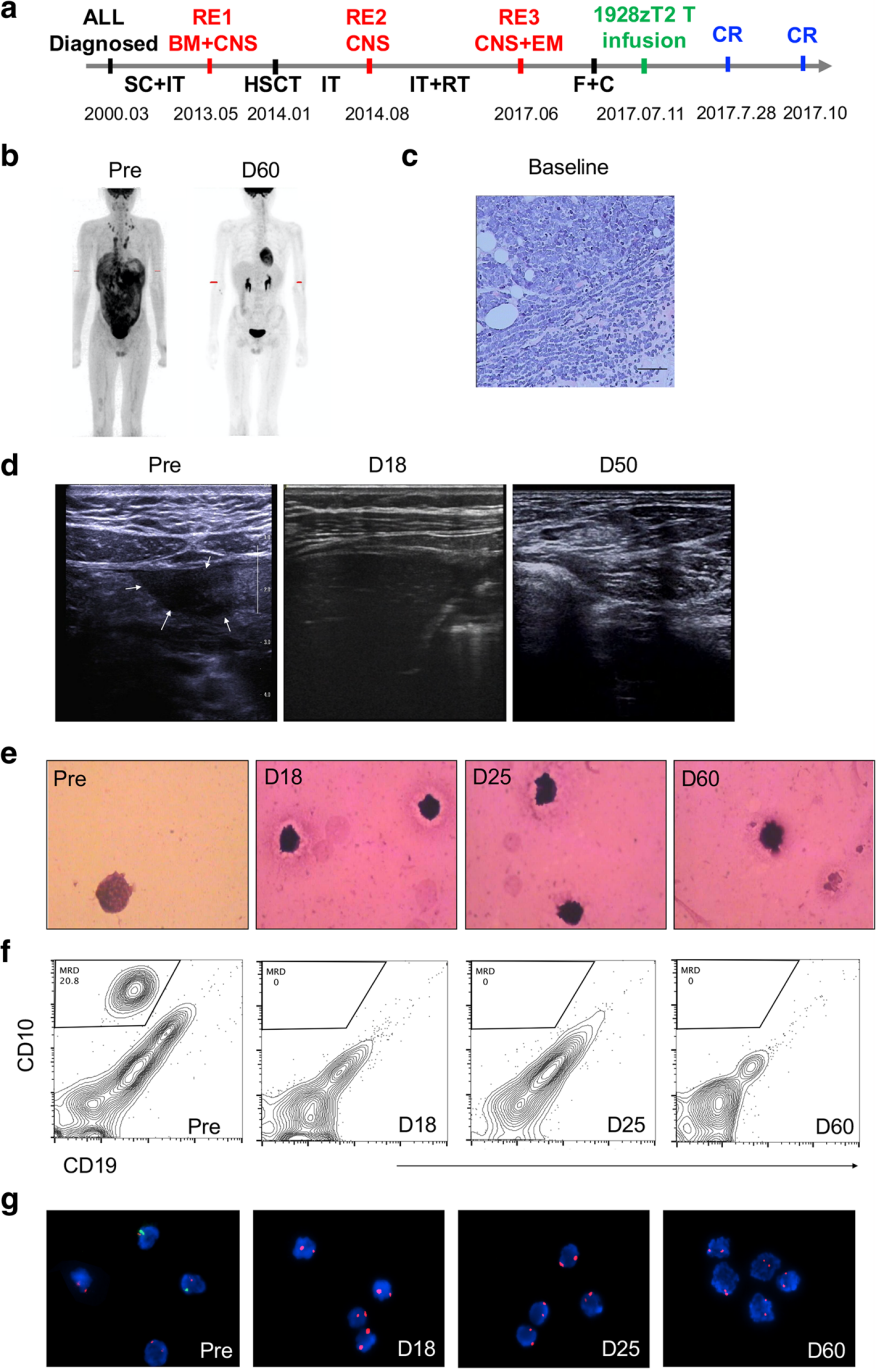
The 1928zT2 T cells exhibited potent anti-leukemia effect in patient 3. **a** shows the development and therapeutic process of this ALL patient with CNS-3 status and extramedullary involvement. He was diagnosed as ALL in March, 2000. The first recurrence (RE1) involved in BM and CNS, and the second RE2 occurred in CNS again. The third relapse (RE3) was identified in CNS and EM in June, 2017. This patient also received fludarabine (F) and cytarabine (C) daily to achieve lymphodepletion and a split-dose infusion of 1928zT2 T cells (1 × 10^6^ cells/kg) was applied. **b** of the PET/CT data shows multiple organs involved by extramedullary ALL, including almost the entire pleura, peritoneum, pelvic fascia, pancreas, and multiple lymph nodes. Restage of PET/CT on D60 after cells infusion presented that no infiltration of cancer cells into the involved tissues at all. **c** A biopsy of the right-sided supraclavicular node mass at baseline shows the presence of blasts, megakaryocytes, erythroblasts, and myeloid cells, proven to be extramedullary relapse. **d** of B-mode ultrasound on tissues below xiphoid shows an inhomogeneous hypo-echoic mass about 3.0 × 1.9 cm in diameter before cells infusion and disappearance of the mass during the follow-up. **e** shows the typical presence of leukemic blast cells (at least 5 blasts in the whole cytocentrifuged smear) in CSF at baseline (Pre-treatment) and only normal mature lymphocytes was seen at D18 and thereafter. **f** of the flow-cytometric data shows that despite about 20.8% blasts with CD19^+^CD10^+^ phenotype was observed in the WBC from CSF before cells infusion, there were undetectable levels of blasts at several time points after treatment. **g** shows that 50% of the cerebrospinal cells were identified as XY-type recipient cells by FISH, indicating the relapse in the CSF. However, there were 100% recipient cells (XX) in CSF after 1928zT2 T cells infusion, suggested complete eradication of blasts in CSF

In patient 3, the visible tumor mass below xiphoid receded gradually beginning on day 3 and disappeared identified by B-mode ultrasound on day 18, indicating the 1928zT2 T cells started to eradicate B-ALL cells rapidly (Fig. 5d). Two months later, restaging PET-CT also showed complete remission in all extramedullary lesions (Fig. 5b). The patient markedly achieved complete clearance of blasts in CSF and relief of symptoms on day 18 post 1928zT2 T cell infusion (Fig. 5e) and obtained recovery of thrombocytopenia and was entitled to receive the lumbar puncture. No CD19^+^CD10^+^ blasts were detected in CSF (Fig. 5f), and X/Y FISH showed that the remaining recipient cells in CSF were 100%XX from the donor (Fig. 5g), suggesting a complete replacement by donor cells and remission in CSF. Collectively, 1928zT2 T cells exhibited potent anti-leukemia effects in these three ALL patients with extramedullary involvement.

### In vivo expansion of 1928zT2 T cells

**Fig. 6:**
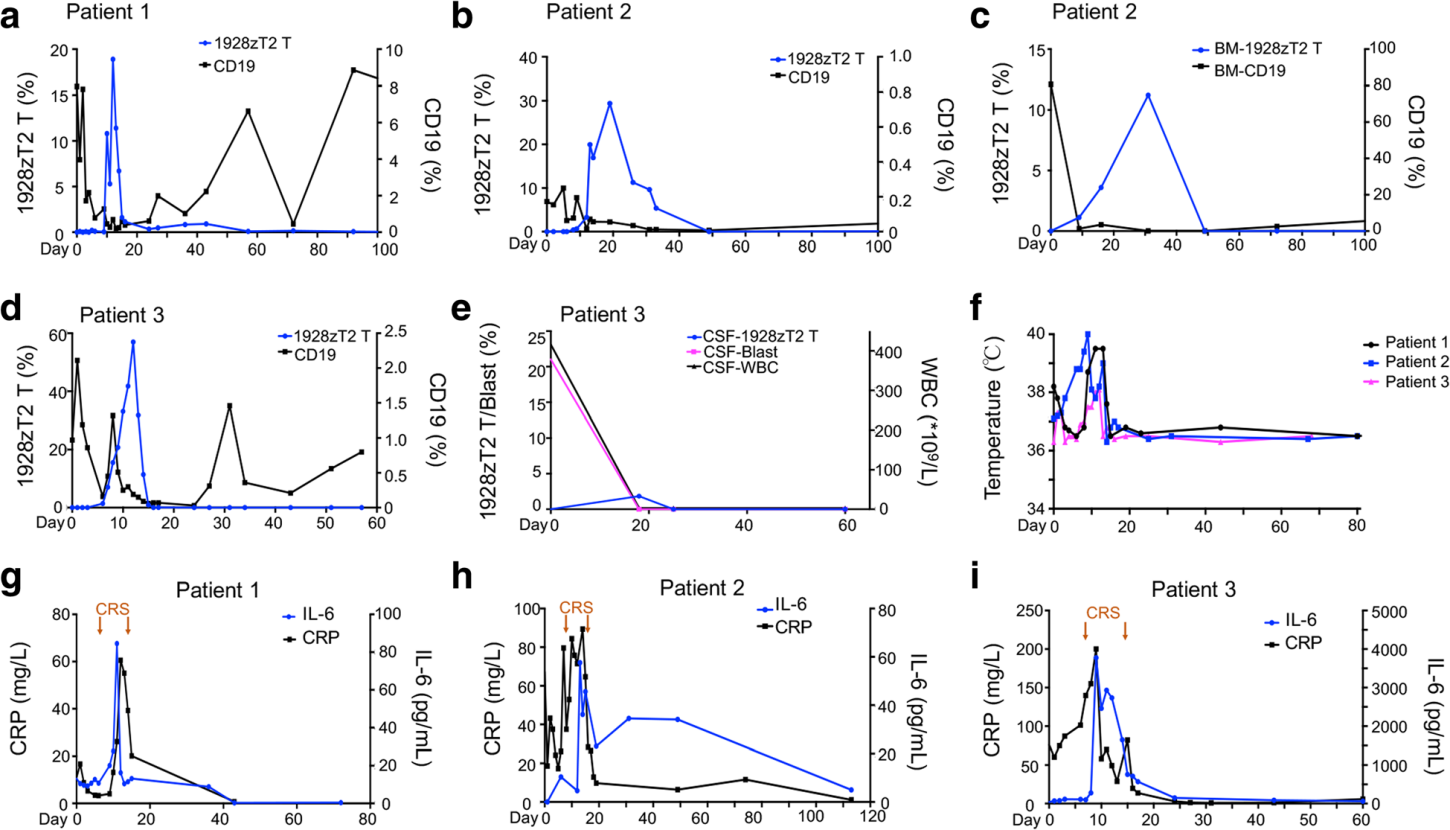
The expansion of 1928zT2 T cells and relevant clinical changes in these patients. **a** The results of flow-cytometric GFP-positive cells analysis in patient 1 showed the 1928zT2 T cells were expanded significantly which began on day 5, peak on day 12, and disappeared after day 92. The CD19-positive cells in periphery blood were dropped quickly within 10 days to the undetectable level and increased again. **b** Patient 2 presented with the presence and elevation of GFP+1928zT2 T cells in the periphery blood from day 10 to day 50. The CD19+ cells were reduced significantly till undetectable. **c** Since the patient 2 had the relapse also in the BM, we found the upregulation of 1928zT2 T cells in the BM, accompanied with eradication of CD19+ cells. **d** The 1928zT2 T cells was expanded significantly on day 12 with 57% of T cells and was undetectable after day 17 in the patient 3. **e** shows the presence of 1928zT2 T cells in CSF from patient 3 with a peak on day 18, indicating the trafficking ability of these cells. Consistently, the blast cells in CSF were undetectable after day 18. **f** shows changes in body temperature after1928zT2 T infusion in these three patients. **g** Patient 1 suffered from grade 2 CRS with fever from day 7 to day 13. IL-6 was elevated slightly with a peak level on day 11, coincided with the occurrence of CRS. The C-Reactive protein (CRP) was also increased. **h** Patient 2 also suffered from CRS with grade 2 from day 9 to day 12 and the expression pattern of IL-6 and CRP was similar to patient 1. **i** For the patient 3, IL-6 was elevated markedly with a peak level on day 10 and decreased after twice usage of tocilizumab, coincided with the occurrence of CRS from day 7 to day 15 (black arrows)

We used the flow-cytometry analysis to detect the GFP-positive 1928zT2 T cells in PBMCs in these three patients. In patient 1, the robust 1928zT2 T cells expansion occurred around day 12 and disappeared by day 92 (Fig. 6a). In addition, CD19+ cells were reduced and became absent within 10 days post the 1928zT2 T cells infusion (Fig. 6a). Then, a continuous rise of IgG level was detected (Fig. 6a), suggesting a slow recovery of B cells in IgG level. For patient 2, 1928zT2 T cells in circulation appeared and began to increase on day 10 after infusion and disappeared on day 50 (Fig. 6b). Similarly, 1928zT2 T cells in BM began to expand on day 10 and retreated on day 50 (Fig. 6c). The flow cytometry analysis results of patient 3 revealed that 1928zT2 T cells in circulation became detectable on day 6 after infusion, and continued increasing to a peak that the percentage of 1928zT2 T cells was as high as 57% in PBMC on day 12, then decreased rapidly and became undetectable after day 17 (Fig. 6d). Since the ALL was relapsed in CNS and extramedullary without BM involvement, MRD- statue in BM was persistently observed. In addition, the 1928zT2 T cells were identified in CSF with 1.84%, indicating the potential ability of these cells traffic to blood-brain barrier (Fig. 6e). No detection of blast cells in CSF also suggested that 1928zT2 T cells exhibited robust immunosurveillance effects in CNS (Fig. 6e).

### Toxicity of 1928zT2 T cells

**Fig. 7:**
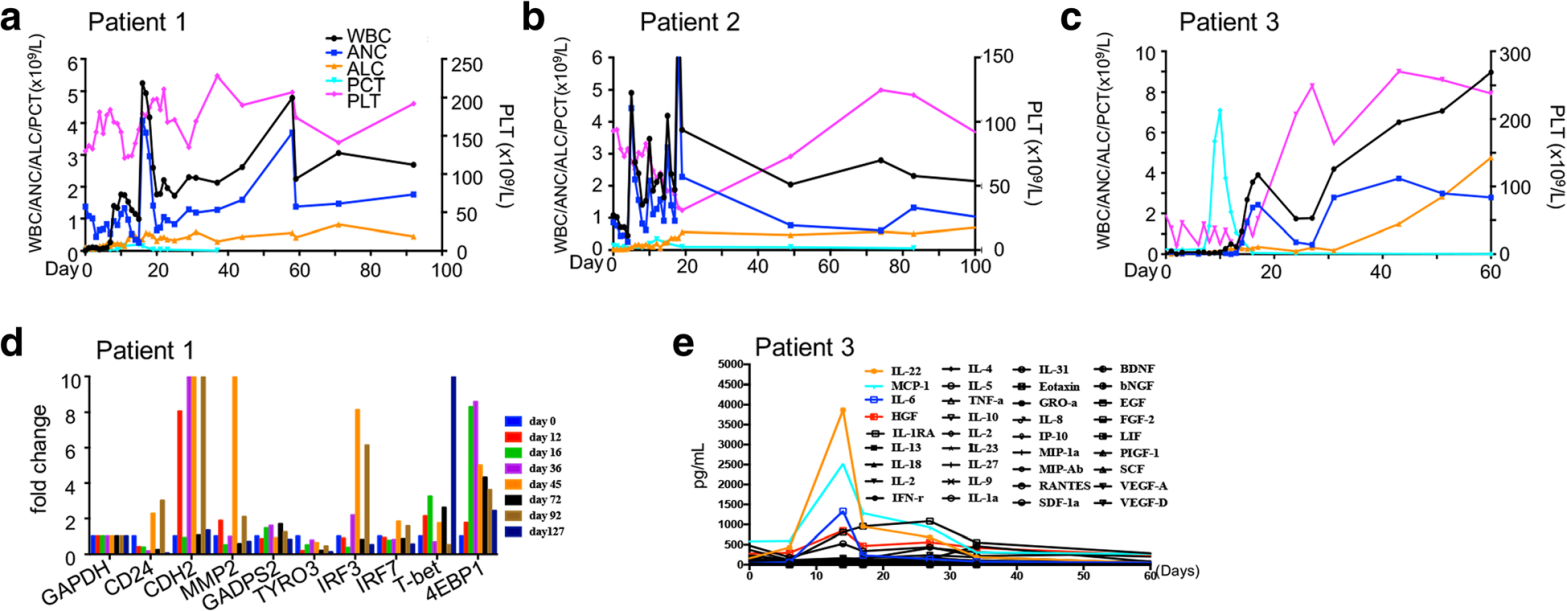
The change of routine blood test and serum cytokines in these patients before and after infusion of 1928zT2 T cells. **a–c** The circulating white blood cells count (WBC), absolute neutrophil count (ANC), absolute lymphocyte count (ALC), and platelet count (PLT) increased after treatment, indicating the recovery of routine blood test results. These blood test maintained in a relatively stable level during the follow-up in these three patients. **d** The qPCR data revealed the expression pattern of the cell adhesion and pro-inflammatory genes in patient 2. **e** shows the cytokine levels in the serum from patient 3 at the indicated time points after1928zT2 T cells infusion. The expressions of IL-22, MCP-1, and HGF presented as the same pattern with IL-6. The IFN-α, IL-17A, GM-CSF, IL-1β, IL-12, IL-7, and IL-15 were below the limits of detection at indicated time points and the other 34 cytokines showed little changes

The 1928zT2 T cells infusion was generally well tolerated in all these three patients. No immediate adverse side effects, episodes of tumor lysis syndrome, autoimmune disorder, and Graft-versus-host disease were observed. However, the patient 1 experienced grade 2 CRS on day 7 post infusion, with fever and left breast pain (Fig. 6g). Her clinical presentation was resolved spontaneously on day 13. The level of IL-6, the main contributor of the CRS [16], increased to a peak on day 11 and decreased sharply on day 12, before the CRS remised (Fig. 6g). We also found the level of C reactive protein (CRP), a common inflammation marker [17], followed a similar pattern of IL-6 (Fig. 6g). She experienced a transient neutropenia on day 14 and day 15, and no other changes in hemogram were observed (Fig. 7a). Patient 2 also experienced CRS with grade 2 between day 9 and day 12, when the levels of IL-6, CRP, and PCT were significantly increased (Figs. 6h and 7b). The patient suffered fever and bone pain. This significant but transient toxicity resolved spontaneously. Patient 3 was diagnosed with grade 3 CRS. He started to have a persistent fever on day 7 and emerged refractory hypotension on day 9, which was alleviated after administrating two usages of tocilizumab (4 mg/kg/d), and was resolved on day 15 (Fig. 6i). The level of IL-6 reached a peak on day 9 concurrently (Fig. 6i) and then declined along with the administration of tocilizumab. The variation tendency of CRP was also accompanied. This patient had an increase in absolute neutrophil count, absolute lymphocyte count, and platelets from the baseline due to the lymphodepletion regime (Fig. 7c). These elevations were also coincident with the expansion of 1928zT2 T cells, suggesting that CAR T cells play a role in the recovery of blood picture.

Several cell adhesion and inflammation genes of patient 1 were also monitored by qPCR, and the increased expression pattern is shown in Fig. 7d. The patient was in a relatively stable state without any additional support. The serum cytokines of patient 3 were monitored using luminex assays, and the levels of IL-22, MCP-1, IL-6, and HGF increased prominently from day 6 to day 17 (Fig. 7e), coincident with the expansion of 1928zT2 T cells in the blood. The concentrations of other 34 tested cytokines including IFN-γ, IL-2, TNF-a, and IL-10 did not change much (Fig. 7e). None of these three patients presented neurotoxicity evidence.

## Discussion

Extramedullary involvement in ALL remains a therapeutic challenge [18]. Although chemotherapy, radiation, surgical resection, and allo-HSCT are all modalities that can be incorporated into the therapy of EM-ALL, the optimal treatment remains unclear and these regimens would induce substantial short-term and long-term toxicities [19, 20]. Until recently, there has been no define therapy for these patients who probably have poor outcomes [21]. We report three extramedullary relapse/refractory B-ALL patients achieved a complete remission by 1928zT2 T cells infusion, which implied the CAR T cells possess a potentially efficacy on EM-ALL relapsed or refractory. The three patients in this study had multiple relapses even though they had received chemotherapy, allo-HSCT, and/or radiation prior to CAR T cells infusion. Our results raise the prospect that 1928zT2 T cells infusion is able to rapidly eradicate extramedullary leukemia without any uncontrolled toxicities.

Notably, 1928zT2 T cells used in this study were incorporated with TLR2 domain, which showed improved anti-leukemia efficacy of CARs [12, 22]. These 1928zT2 T cells not only could eradicate the CD19-expressing NALM6 cells and K562 cells specifically and potently in vitro, they could significantly promote the remission of NALM-6 subcutaneous tumors ex vivo, indicating the TLR2 incorporation enhanced the anti-leukemia effects of CAR T cells. Of note, the patient 1 was infused with a dose of 1928zT2 T cells as low as 5 × 10^4^ cells/kg and still achieved CR. As we know, the receptor of TLR2, an essential bridge of innate and adaptive immunity, has the potential to mobilize antigen-presentation response and promote specific activation and expansion of T cells, exhibiting critical regulation of overall immune responses [23]. Previous studies have reported that TLR2 costimulation would promote adhesion and immune synaptic signaling between CAR T cells and target cells [24]. In this study, we also found the obvious elevations of MCP-1 [25] and HGF [26], the critical chemokines for directing migration of lymphocytes, in the patient 2 after 1928zT2 T cells infusion, indicating the possible involvement of TLR2 in adhesive signaling. In addition, the elevations of MCP-1 and HGF were coincident with the expansion and trafficking of 1928zT2 T cells, suggesting possible roles of MCP-1 and HGF in regulating the trafficking of 1928zT2 T cells. Further studies are required to investigate the mechanisms how TLR2 incorporation promote CAR T cells to infiltrate into tumors and disrupt tumor microenvironment, which are the major challenges for treating CNS and extramedullary leukemia.

1928zT2 T cells infusion may overcome many limitations of conventional therapies. For instance, CNS is regarded as a pharmacological sanctuary for leukemia cells [27]. However, 1928zT2 T cells could traffic to the CNS and eradicate leukemia here without any overt CNS toxicities. Although some studies reported that CAR T cells emerging in CSF are associated with development of neurotoxicity [28], we did not find any evidence of neurotoxicity in patient 3. It was reported that the Phase II ROCKET trial of JCAR015 (19-28z CAR T cells) was halted for ALL due to the development of sever cerebral edema and subsequent death of several patients, which was associated with rapid expansion and exhaustion of CAR T-cells with CD28. Until now, there was no direct evidence of possible causal relationships of CD28 incorporation and neurotoxicity of CAR T-cells [29]. It is speculated that CAR T cells incorporating 4-1BB are more persistent and less likely to undergo exhaustion than those incorporating CD28, contributing to improved tolerability [30, 31]. However, from the primary data of nine patients using 1928zT2 T cells with CD28 in our phase I clinical trial, no patients suffered from cerebral edema during the follow-up. Overall, the well tolerance and efficacy of 1928zT2 T cells in this study provides a promise for CNS-3 status leukemia patients, who were ineligible for CAR T cells before, though more patients are needed to be recruited in the clinical trial.

In addition, the incorporation of CD28 and TLR2 costimulatory domains in this study was different from the other third-generation CARs, which incorporate two costimulatory domains such as CD28 and 4-1BB or OX-40. These additional costimulatory signals could improve the expansion of CAR T cells. Most third-generation CAR T cells would improve cytokine secretion due to the productive T cell-stimulation and probably induce the severe CRS in patients. At present, most available CAR T cells usage in clinical were the second-generation ones and no direct comparison between these third generation CARs was reported. In this study, it was of note that the 1928zT2 T cells infusion just induced grade 2 or 3 CRS and these patients recovered spontaneously or after treatment with tocilizumab. Although we observed an onset of several cytokines elevations in parallel with robust T cell expansion in these patients, which was consistent with previous studies suggesting some degree of CRS is probably necessary for CAR T cells efficacy [32], the rest 34 tested cytokines did not fluctuate, indicating that the limited cytokine secretion was possibly related with the low toxicity of 1928zT2 T cells.

## Conclusions

Our study demonstrated that the 1928zT2 T cells rapidly and reliably eradicated extramedullary and CNS leukemia and were well tolerated in these three ALL patients. Our results therefore suggested that adoptive transfer of 1928zT2 T cells has great potential to be used to treated ALL patients with extramedullary and CNS leukemia in the future.

## Abbreviations

ALL: Acute lymphoblastic leukemia
allo-HSCT: Allogeneic hematopoietic stem cell transplantation
CAR-T cells: Chimeric antigen receptor T cells
CNS: Central nervous system
CRS: Cytokine releasing syndrome
IT: Intrathecal chemotherapy
SC: Systemic chemotherapy
TLR2: Toll-like receptor 2
