# Correction to: A novel generation 1928zT2 CAR T cells induce remission in extramedullary relapse of acute lymphoblastic leukemia

**DOI:** 10.1186/s13045-019-0816-4

**Published:** 2019-11-20

**Authors:** Jianyu Weng, Peilong Lai, Le Qin, Yunxin Lai, Zhiwu Jiang, Chenwei Luo, Xin Huang, Suijing Wu, Dan Shao, Chengxin Deng, Lisi Huang, Zesheng Lu, Maohua Zhou, Lingji Zeng, Dongmei Chen, Yulian Wang, Xiaomei Chen, Suxia Geng, Robert Weinkove, Zhaoyang Tang, Chang He, Peng Li, Xin Du

**Affiliations:** 1 Department of Hematology, Guangdong General Hospital, Guangdong Academy of Medical Sciences, Guangzhou, 510080 China; 2 Key Laboratory of Regenerative Biology, South China Institute for Stem Cell Biology and Regenerative Medicine, Guangzhou Institutes of Biomedicine and Health, Chinese Academy of Sciences, Guangzhou, 510530 China; 3 Guangdong Provincial Key Laboratory of Stem Cell and Regenerative Medicine, South China Institute for Stem Cell Biology and Regenerative Medicine, Guangzhou Institutes of Biomedicine and Health, Chinese Academy of Sciences, Guangzhou, 510530 China; 4 Department of PET Center, Guangdong General Hospital and Guangdong Academy of Medical Sciences, Guangzhou, 510080 China; 5 The Malaghan Institute of Medical Research, Wellington, New Zealand; 6 Guangdong Zhaotai InVivo Biomedicine Co. Ltd., Guangzhou, 510000 China; 7 Hunan Zhaotai Yongren Medical Innovation Co. Ltd., Changsha, 410000 China; 8 State Key Laboratory of Ophthalmology, Zhongshan Ophthalmic Center, Sun Yat-Sen University, Guangzhou, 510060 People’s Republic of China

**Correction to: J Hematol Oncol**

**https://doi.org/10.1186/s13045-018-0572-x**

The original article [1] contains an error in authorship whereby author, Robert Weinkove’s name is mistakenly inverted. The configuration noted in this Correction article should be considered instead along with author’s updated affiliation.
